# PD-1/PD-L1 Correlates With the Efficacy of the Treatment of Concurrent Chemoradiotherapy in Cervical Cancer

**DOI:** 10.3389/fonc.2022.858164

**Published:** 2022-05-10

**Authors:** Hanqun Zhang, Shisheng Tan, Chunju Fang, Qi Zhang, Xue Cao, Yuncong Liu

**Affiliations:** Department of Oncology, Guizhou Provincial People’s Hospital, Guizhou, China

**Keywords:** cervical cancer, programmed cell death 1, programmed cell death 1 ligand, concurrent chemoradiotherapy, prognosis

## Abstract

**Background:**

Cervical cancer (CC) is the third most common cancer worldwide, with high mortality rates. The programmed cell death 1 (PD-1)/(PD-1 ligand) PD-L1 has been reported to be an effective indicator in cancer development. In this study, we aim to explore the role of PD-1/PD-L1 in the evaluation of concurrent chemoradiotherapy (CCRT) efficacy and prognosis in CC patients.

**Methods:**

We included 55 CC patients in this study. Immunohistochemistry and flow cytometry were employed to detect the expression of PD-1, T_reg_ cells, CD8, and CD68 in tumor tissues, and the contents of PD-1^+^ CD8^+^ T cells, PD-1^+^ CD4^+^ T cells, and PD-1^+^ T_reg_ cells in the peripheral blood. The relationships of these indexes with CCRT efficacy were measured by Spearman correlation analysis, overall survival (OS), and disease-free survival (DFS) of patients were analyzed by Kaplan–Meier estimator, and the diagnostic values of these indexes in CC were assessed by a receiver operating characteristic (ROC) curve.

**Results:**

The clinical effectivity rate of CCRT was 89.10%. The positive expressions of PD-L1, T_reg_ cells, PD-1^+^ CD8^+^ T cells, PD-1^+^ CD4^+^ T cells, and PD-1^+^ T_reg_ cells were reduced after CCRT, while the CD8 and CD68 increased. All 7 indexes had diagnostic values in evaluating CCRT efficacy and were considered the influencing factors of OS, DFS, and the prognosis of CC patients.

**Conclusion:**

These findings indicate that PD-1/PD-L1 may be a potential indicator for the efficacy evaluation of CCRT and the prognosis of CC. This study may offer potential targets for CC treatment.

## Introduction

Cervical cancer (CC) is one of the gynecological cancers and the fourth leading cause of cancer-related mortality among women worldwide (1). It is the third most commonly diagnosed cancer in the world and the second most frequently diagnosed cancer among women in developing countries (2). Every year, more than 530,000 women are diagnosed with CC throughout the world, which accounts for over 275,000 deaths (3). After applying PAP smear and human papillomavirus (HPV) vaccination, early diagnosis of cervical cancer and diagnosis of CINs have increased. However, due to the influence of social, economic, and cultural factors, over 70% of CC cases diagnosed in developing countries are locally invasive or metastatic, and the diagnosis of early-stage CC is difficult, which leads to the high mortality of CC (4). It is widely accepted that long-term HPV infection in the uterine cervix is the major cause of the occurrence and development of CC (5). At present, the clinical treatment of CC includes radiotherapy, chemotherapy, and surgery; however, the efficacy of treatment varies significantly among patients, and the prognosis is still not satisfactory (6). In addition, the 5-year survival rate of patients with metastatic CC is only about 50% (4). Recent studies have demonstrated that many genes are potential prognostic markers in CC (7). Thus, the main objective of this study is to evaluate the value of PD-1/PD-L1 for the diagnosis and prognosis of CC.

The programmed cell death 1 (PD-1), as a member of the B7/CD28 group in the immunoglobulin superfamily reporters, has another name, CD279, and two ligands, PD-L1 (CD274) and PD-L2 (CD273) (8). PD-1 has been shown to appear on a variety of immune cells, including activated T cells, myeloid cells, B cells, natural killer cells, thymocytes, and CD4^−^ CD8^−^ thymocytes (9). The potential role of PD-1 in maintaining peripheral tolerance by negatively regulating the responses of T cells to antigen stimulation has been reported in previous research (10). A recent study confirmed that the PD-1 is overexpressed in the tumor-reactive population at primary and secondary tumor sites, and its inhibition significantly improves antitumor immunity and suppresses tumor growth (11). Moreover, researchers have identified PD-1 as a tumor growth receptor that promotes tumorigenesis in melanoma cells (12). Additionally, the expression of PD-L1 can be investigated as a potential biomarker for the immunotherapeutic response, and the blockade of PD-1 and PD-L1 interaction has proved significant in anticancer activity (13). The interaction between the PD-1 and PD-L1 exerts a critical function on the activation and expansion of autoreactive T cells by reducing T-cell responses (14). Based upon the above, this study aims to explore the involvement of PD-1/PD-L1 expression in the evaluation of efficacy and prognosis of CC.

## Material and Methods

### Ethics Statement

The study was approved by the Institutional Review Board of Gui Zhou Provincial People’s Hospital (2016085). Written informed consent was obtained from all patients or their parents or guardians.

### Study Subjects

From January 2013 to December 2015, 55 patients with CC were included in this study. According to the International Federation of Gynecology and Obstetrics (FIGO) staging system (2009), the tumors were in stage IIb~IIIb, and all tumors were pathologically confirmed as CC. All patients were first struck CC and got treatment for the first time. None received chemoradiotherapy before this study. The Karnofsky performance scale (KPS) scores were ≥70 and the Eastern Cooperative Oncology Group (ECOG) scores were 0~2. Patients were excluded when they are under any of the following conditions: with malignancy in other parts of the body; with active diseases, infectious diseases, and dominant syphilis; in the hepatitis B active period; incapable of receiving chemoradiotherapy because of other diseases in other important organs; or being pregnant. The 55 patients were from 25 to 74 years of age, with a mean age of 53, and the mean KPS score was (78.41 ± 3.18). All patients received examinations before and after therapy, including blood cell analysis, examination of liver and kidney, determination of serum glucose, coagulation test, detection of C-reactive protein and multiple tumor markers, classification of lymphocyte subsets in the peripheral blood, preoperative immunotherapy, urine analysis + examination of urinary sediment, stool examination + occult blood test, and electrocardiographic examination.

### CCRT

Before and after the therapy, all patients received examinations, including routine blood tests, coagulation tests, electrocardiogram examination, examination of the liver and kidney, and computed tomography (CT)/magnetic resonance imaging (MRI) of the pelvic cavity. A combined treatment of external beam radiotherapy and high-dose-rate intracavity brachytherapy was performed for all patients. The external beam radiotherapy was using intensity-modulated radiation therapy (IMRT) (ELEKTA Synergy). ELEKTRA fields were set routinely, and the whole pelvic irradiation was conducted for 5 weeks at 5 times a week, one field each time, and 2 Gy total dose (DT) for each field; the total dose was 50 Gy. The whole pelvic irradiation was changed into the pelvic external beam radiotherapy for four fields in the third week, and brachytherapy was conducted using a 192Ir brachytherapy machine (Nucletron microSelection V3). The ELEKTRA therapy was conducted 6 times once a week and 6 Gy (EQD2 dose conversion to regular dose: 8 Gy) in A point each time and the total dose was 36 Gy (EQD2 dose conversion to regular dose: 48 Gy). Chemotherapy was started at the same time as radiotherapy, and the specific procedures were as follows: 40 mg/m^2^ cisplatin as a single agent was injected into the vein, and the first course of cisplatin administration started on the first day of radiotherapy and lasted 5 weeks. A total of 2 ml of blood anticoagulated with ethylenediaminetetraacetic acid (EDTA) of patients before and after therapy (before the last loading) was collected for detection, respectively; 5 ml of blood nonanticoagulated with EDTA was collected, and serum was isolated and stored at −70°C for subsequent detection. The cancer tissues of patients were collected by operation, and the samples were fixed with formaldehyde.

### Immunohistochemistry

The CC tissues were fixed in 10% formaldehyde, embedded in paraffin, and sliced into 4 μm sections. The sections were baked in a 60°C incubator for 1 h, conventionally dewaxed by xylene, dehydrated with gradient alcohol, incubated in 3% H_2_O_2_ (Sigma-Aldrich Chemical Company, St Louis, MO, USA) at 37°C for 30 min, and washed with phosphate-buffered saline (PBS) (Desen, China). The sections were then boiled in 0.01 M citric acid buffer at 95°C for 20 min, cooled to room temperature, and washed with PBS. The primary antibody programmed death-ligand 1 (PD-L1) (15 µg/ml) monoclonal antibody (BD Biosciences, USA), transcription factor forkhead box P3 (FOXP3) primary antibody working liquid (1:100, BD Biosciences, USA), CD8 rat anti-human monoclonal antibody (1:150, BD Biosciences, USA), and CD68 antibody (BD Biosciences, USA) were added into sections respectively at 4°C overnight. After, the sections were washed with PBS, colored with diaminobenzidine (DAB) (Desen, China), counterstained with hematoxylin (Shanghai Bogoo Biological Technology Co. Ltd., Shanghai, China), and mounted. Ten discontiguous fields were observed under a high-power microscope (×400), and the percentage of positive cells to total cells was evaluated. The median of these figures was used as a cutoff value to divide the results of high expression and low expression. Cell types were then positively stained as follows: PD-L1, brown or tan granules in the cytomembrane and cytoplasm; T_reg_, tan granules in the nucleus; CD8, yellow and brown granules in the cytomembrane and cytoplasm; CD68, tan granules in the cytoplasm.

### Flow Cytometry

To extract peripheral blood mononuclear cells (PBMC), the peripheral venous blood of patients was drawn before and after therapy, treated with anticoagulant, mixed with PBS (1:1), added to lymphocytes separation medium (1:1), and centrifuged at room temperature for 20 min (1,500×*g*). The PBMCs were placed in a centrifuge tube, centrifuged for 5 min (800×*g*) with 10 ml PBS, and washed 3 times for subsequent experiments. Antibodies were added as follows: tube 1: CD3 (PerCP), CD4 (FitC), CD8 (APC), and CD279 (PE) (BD Biosciences, USA); tube 2: CD4 (FitC), CD25 (APC), CD127 (PE-CY7), and CD279 (PE) (BD Biosciences, USA) and 30 μl whole blood, then the mixture was shaken and protected from light for 20 min. The blood samples were shaken before being added with 600 μl hemolysin, protected from light, centrifuged for 5 min (402×*g*), added with 1 ml PBS, and centrifuged again. CD8^+^ and CD4^+^ were used for gate setting, cells stained with immunoglobulin G (IgG) were used as boundaries for FL-1, FL2, FL-3, FL4, and the results were analyzed by CELLQuest software.

### Follow-Up

Outpatient follow-up and telephone follow-up were conducted once every 3 months in the first year after therapy. Examinations including gynecological examination, ThinPrep cytological test (TCT), and CT/MRI of the abdominopelvic cavity were conducted for assessment. Until the cutoff time in December 2016, there was no loss to follow-up in 55 patients, so the rate of follow-up was 100%.

### Evaluation of Efficacy and Adverse Effect

The efficacy of chemoradiotherapy was evaluated according to the response evaluation criteria in solid tumors (RECIST version 1.1) (15): (1) complete response (CR), all target lesions disappeared, and the diameter of lymph node was reduced below 10 mm; (2) partial response (PR), the total of the longest diameters of target lesions reduced more than 30%; (3) stable disease (SD), the total of the longest diameters of target lesions reduced less than 30% or increased less than 20%; (4) progressive disease (PD), the total of the longest diameters of target lesions increased more than 20%, and the target lesions absolutely increased more than 5 mm, or one or more new target lesions appeared. The effective rate = (CR + PR)/*n* × 100%.

The adverse effect was divided into recent and long-term toxicity reactions. The recent toxicity reaction occurs within 3 months from the start of therapy to the end of it, while that occurs 3 months later is regarded as a long-term toxicity reaction. The criteria of assessment are based on the National Cancer Institute-Common Toxicity Criteria (NCI-CTC version 2.0) and Radiation Therapy Oncology Group (RTOG)/European Organization for Research and Treatment of Cancer (EORTC) (16, 17).

One year after the therapy, the overall survival (OS) and disease-free survival (DFS) were observed. OS is the period from the start of this study to the last follow-up or death; DFS is the period from the start of this study to the time in which cancer tissues recrudesced or metastasized or the last follow-up.

### Statistical Analysis

Statistical analysis was conducted using SPSS 21.0 (IBM Corp. Armonk, NY, USA). Measurement data were expressed as a mean and standard deviation; the Kolmogorov–Smirnov method was used for the normality test. The *post-hoc* tests were conducted using Dunn’s multiple comparisons test following a Kruskal–Wallis test for data with skew distribution; data differences between two groups with normal distribution and equal variance were analyzed using an independent sample *t*-test, while the differences in one group were analyzed using paired *t*-test. Data with normal distribution and heterogeneous variance were adjusted using Welch’s *t*-test. Measurement data were expressed as (%) and analyzed by the Chi-square test. The survival curve was drawn by using Kaplan–Meier estimator, and the differences were determined using the Log-rank test. The clinical diagnostic value of each index was assessed using the receiver operating characteristic (ROC) curve and cutoff point selection. *p* < 0.05 was considered statistically significant.

## Results

### Higher Clinical Effective Rate Shown in Patients With CC After CCRT

The efficacy of chemoradiotherapy and its adverse effect were assessed according to RECIST version 1.1, NCI-CTC version 2.0, and RTOG/EORTC. The clinicopathological features of all 55 patients are shown in **Supplementary Table S1**. The number of patients with CR, PR, SD, and PD was 35, 14, 4, and 2, respectively, and the effective clinical rate was 89.10%. A total of 18 patients had a recent toxicity reaction, and 12 patients had long-term toxicity reactions; the adverse effect manifested as myelosuppression of leukocytes and neutrophils, nausea and vomiting, and diarrhea.

### CCRT Inhibits Positive Expression of PD-L1 and T_reg_ But Promotes That of CD8 and CD68

**Figure 1:**
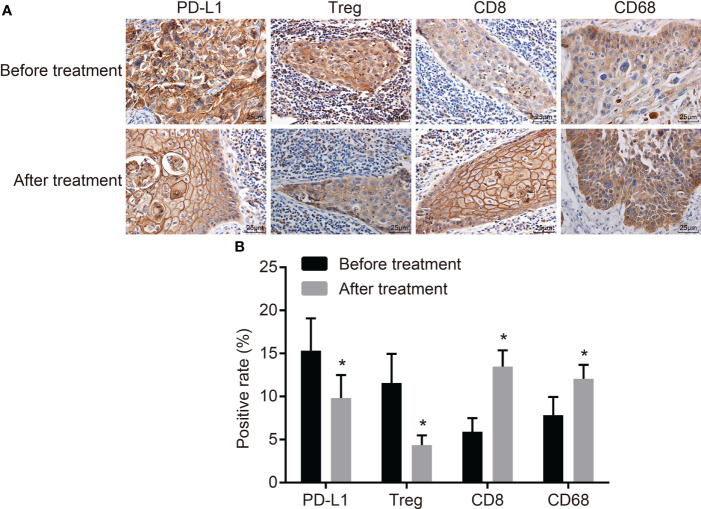
CCRT suppresses PD-L1 and T_reg_-cell expressions but improves CD8 and CD68 expressions. **(A)** Positive expressions of PD-L1 and T_reg_ are decreased, CD8 and CD68 are increased before and after CCRT is detected by immunohistochemistry (the original magnification is ×400). **(B)** Positive expression rates of PD-L1 and T_reg_ cells were decreased, but that of CD8 and CD68 elevated after CCRT; expression rates were the measurement data, and were expressed as mean and standard deviation, paired *t*-test was used for intragroup comparison; ^*^p < 0.05, compared with the figures after CCRT. CCRT, concurrent chemoradiotherapy; PD-L1, programmed death ligand 1.

Immunohistochemistry was conducted to detect the expression of PD-L1, T_reg_, CD8, and CD68 (**Figure 1A**). After CCRT, the brown and tan granules of PD-L1 in the cytomembrane and cytoplasm and the tan granules of T_reg_ in the nucleus were reduced, while the tan granules of CD8 in the cytomembrane and cytoplasm and the tan granules of CD68 in the cytoplasm were increased. These results indicated that after CCRT, the positive expression rate of PD-L1 and T_reg_ cells was significantly lower, while the positive expression rate of CD8 and CD68 was higher than before (*p* < 0.05) (**Figure 1B**).

### PD-L1, T_reg_ Cell, CD8, and CD68 Expressions Are Associated With CCRT Efficacy

**Figure 2:**
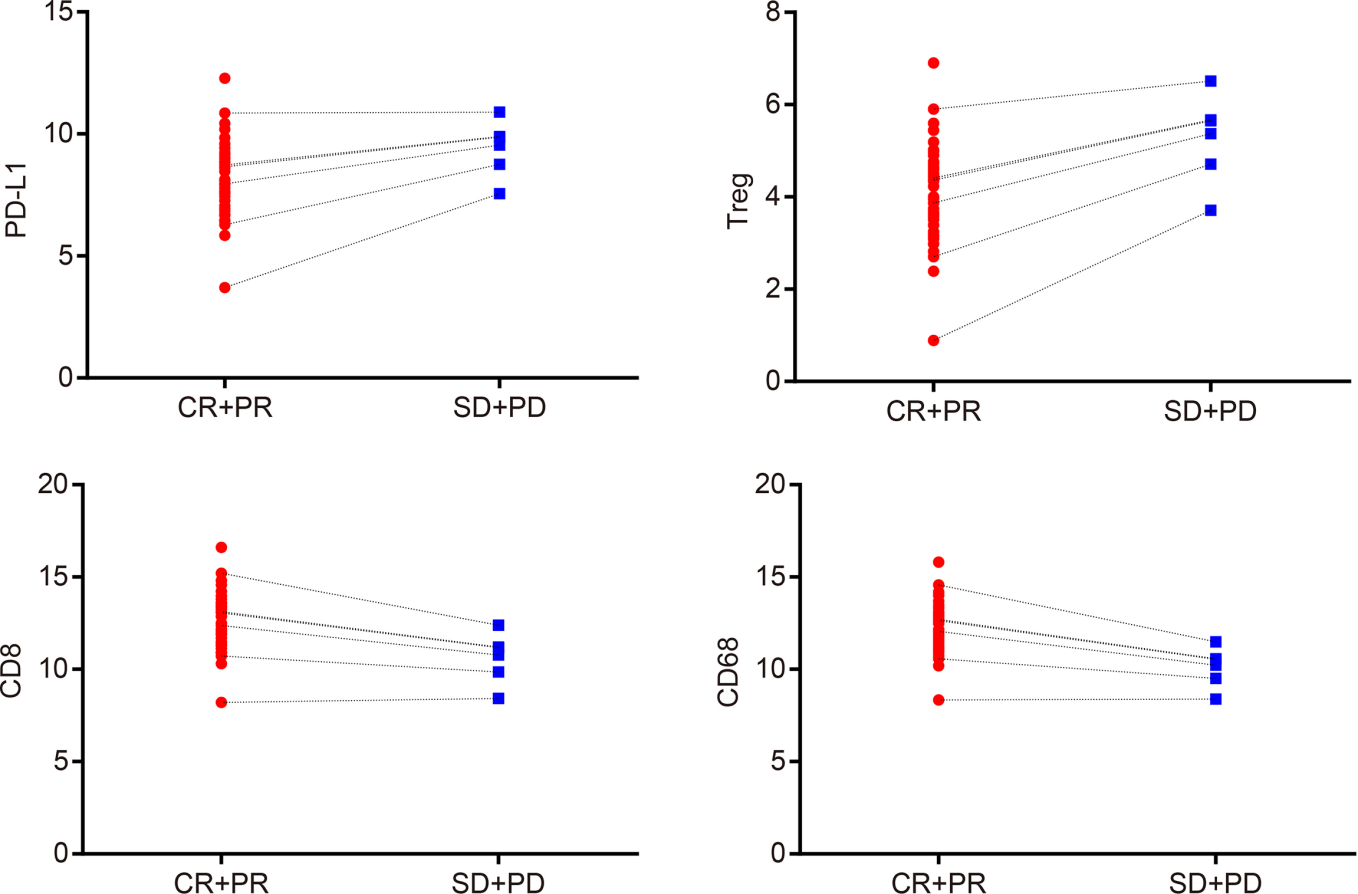
PD-L1, T_reg_ cells, CD8, and CD68 expressions are associated with the efficacy of CCRT. Spearman’s correlation analysis indicates that expressions of PD-L1, T_reg_ cells, CD8, and CD68 are correlated with curative effect; CR + PR: *n* = 49; SD + PD: *n* = 6; CR, complete response; PR, partial response; SD, stable disease; PD, progressive disease; PD-L1, programmed death ligand 1; CCRT, concurrent chemoradiotherapy.

**Figure 3:**
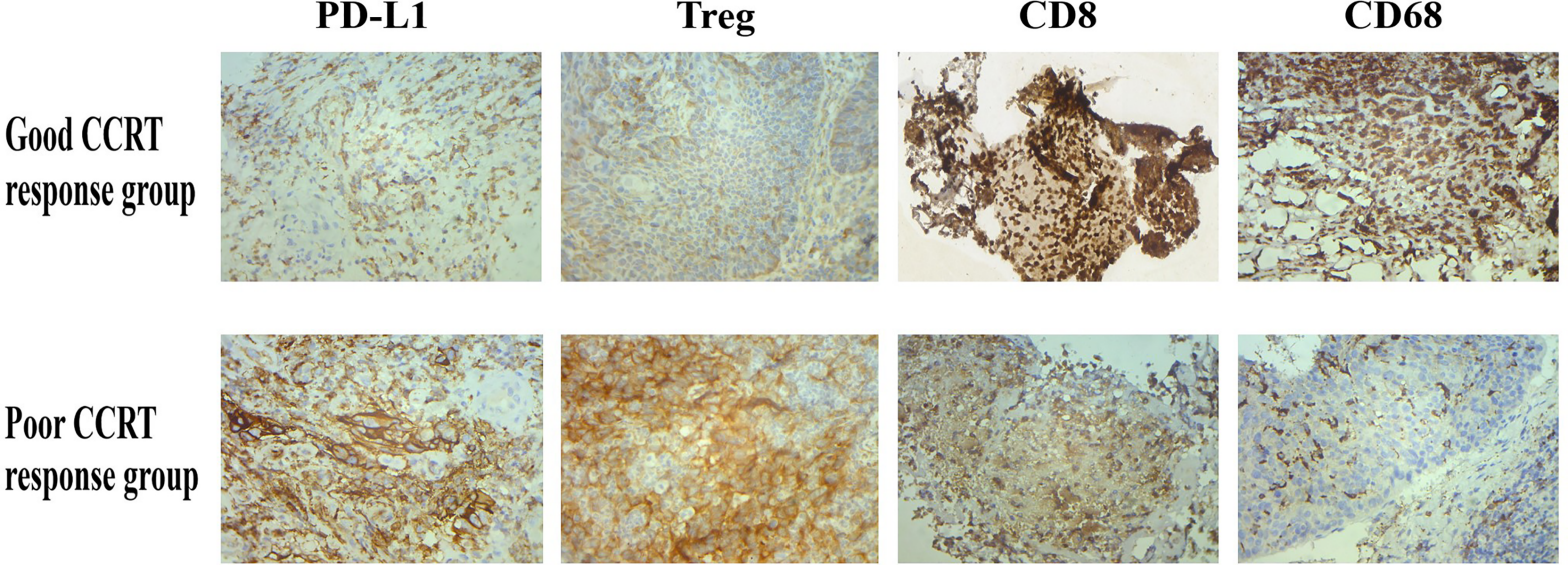
After CCRT, PD-L1 and T_reg_ expressions were inhibited in tumor tissues of patients with good treatment response, while CD8 and CD68 expressions were increased; the above situation was reversed in patients with poor treatment response.

The Spearman correlation analysis was used to analyze the correlation coefficients of PD-L1, T_reg_ cell, CD8, and CD68 expressions and curative effect (**Supplementary Table S2**) (*r* = 0.829, *p* = 0.042; *r* = 0.943, *p* = 0.005; *r* = 0.829, *p* = 0.042; *r* = 1.000, *p* < 0.001 (**Figure 2**). In addition, the expression of PD-L1, T_reg_, CD8, and CD68 was associated with therapeutic efficacy in immunohistochemistry (**Figure 3**). These results indicated that PD-L1, T_reg_ cell, CD8, and CD68 expressions were related to the efficacy of CCRT.

### CCRT Reduces Expressions of PD-1^+^ CD8^+^, PD-1^+^ CD4^+^, and PD-1^+^ T_reg_ in T Cells in the Peripheral Blood

**Figure 4:**
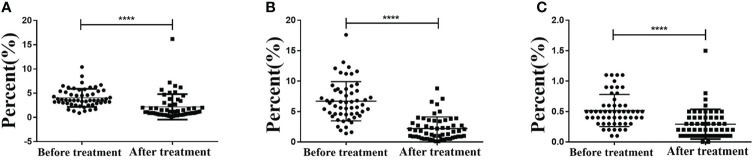
CCRT lessens PD-1^+^ CD8^+^ T cells, PD-1^+^ CD8^+^ T cells, PD-1^+^ CD4^+^ T cells, and PD-1^+^ T_reg_ cells in peripheral blood detected by flow cytometry. **Panel A**, PD-1^+^ CD8^+^ T cells in peripheral blood decreased after CCRT detected by immunohistochemistry; **Panel B**, PD-1^+^ CD4^+^ T cells in peripheral blood decreased after CCRT detected by immunohistochemistry; **Panel C**, PD-1^+^ Treg cells in peripheral blood decreased after CCRT detected by immunohistochemistry; measurement data were expressed as mean and standard deviation and analyzed by paired t test, n = 55; (**** *p*<0.0001), compared with before CCRT; CCRT, concurrent chemoradiotherapy; PD-1, programmed death 1.

Flow cytometry was performed to detect the expressions of PD-1^+^ CD8^+^ and PD-1^+^ CD4^+^ in T cells before and after CCRT. The results showed that the expression of PD-1^+^ CD8^+^ in T cells before and after CCRT was (4.042 ± 1.856) and (2.175 ± 2.632), respectively, and that of PD-1^+^ CD4^+^ was (6.702 ± 3.221) and (2.213 ± 1.907), respectively. The PD-1 expression in T_reg_ cells before and after CCRT was (0.518 ± 0.263) and (0.293 ± 0.244), respectively. These findings demonstrated that after CCRT, expressions of PD-1^+^ CD8^+^ T cells, PD-1^+^ CD4^+^ T cells, and PD-1^+^ T_reg_ cells in the peripheral blood were reduced (*p* < 0.05) (**Figures 4A**–**C**).

### Lower PD-1^+^ CD8^+^ T Cells, PD-1^+^ CD4^+^ T Cells, and PD-1^+^ T_reg_ Cells Represented Higher Efficacy of CCRT

**Figure 5:**
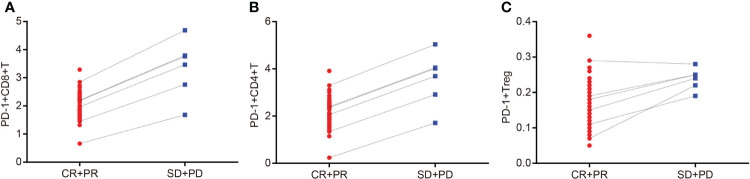
Contents of PD-1^+^ CD8^+^ T cells, PD-1^+^ CD4^+^ T cells, and PD-1^+^ T_reg_ cells are related to the efficacy of CCRT. **Panel A**, Spearman correlation analysis indicates that content of PD-1^+^ CD8^+^ T cells is correlated with efficacy of CCRT; **Panel B**, Spearman correlation analysis indicates that content of PD-1^+^ CD4^+^ T cells is correlated with efficacy of CCRT; **Panel C**, Spearman correlation analysis indicates that content of PD-1^+^ Treg cells is correlated with efficacy of CCRT; CR + PR: n = 49; SD + PD: n = 6 ; PD-1, programmed death 1; CR, complete response; PR, partial response; SD, stable disease; PD, progressive disease; CCRT, concurrent chemoradiotherapy.

The Spearman correlation analysis was used to analyze the correlation coefficients of PD-1^+^ CD8^+^ T cells, PD-1^+^ CD4^+^ T cells, and PD-1^+^ T_reg_ cells with a curative effect, and the results were as follows: *r* = 0.829, *p* = 0.042; *r* = 0.943, *p* = 0.005; *r* = 0.928, *p* = 0.008 (**Supplementary Table S3**; **Figures 5A**–**C**). These results indicated that the contents of PD-1^+^ CD8^+^ T cells, PD-1^+^ CD4^+^ T cells, and PD-1^+^ T_reg_ cells were visibly related to the efficacy of CCRT.

### PD-1/PD-L1 Is Effective for Evaluation of CCRT Efficacy in CC

**Figure 6:**
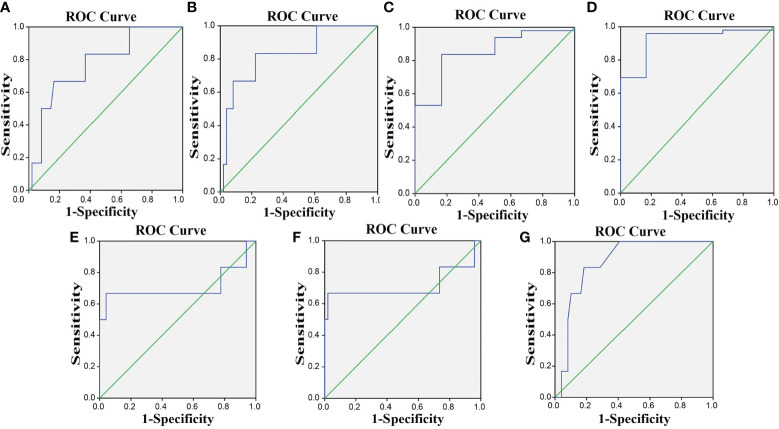
The ROC curves indicate that PD-1/PD-L1 is effective for the efficacy evaluation of CCRT in CC. **(A–G)** The ROC curves for PD-L1, T_reg_, CD8, CD68, PD-1^+^ CD8^+^ T cells, PD-1^+^ CD4^+^ T cells, and PD-1^+^ T_reg_. PD-L1, programmed death ligand 1; PD-1, programmed death 1; CC, cervical cancer; ROC, receiver operating characteristic.

ROC was used to determine the diagnostic effect of PD-L1, T_reg_ cells, CD8, CD68, PD-1^+^ CD8^+^ T cells, PD-1^+^ CD4^+^ T cells, and PD-1^+^ T_reg_ cells. CR + PR represents effective therapy, and SD + PD represents ineffective therapy. The area under the ROC curve (AUC) of the diagnostic effect of PD-L1, T_reg_ cells, and CD8 and CD68 expressions was 0.774, 0.830, 0.850, and 0.922, respectively, and the Youden’s index was 0.503, 0.609, 0.670, and 0.793, respectively. The AUC of the diagnostic effect of PD-1^+^ CD8^+^ T cells, PD-1^+^ CD4^+^ T cells, and PD-1^+^ T_reg_ cells was 0.707, 0.714, and 0.864, and the Youden’s index was 0.626, 0.646, and 0.65, respectively. The sensitivity and specificity of these indexes at the cutoff point are shown in **Supplementary Table S4**. The ROC curve of the diagnostic effect of PD-L1, T_reg_ cells, CD8, CD68, PD-1^+^ CD8^+^ T cells, PD-1^+^ CD4^+^ T cells, and PD-1^+^ T_reg_ cells is shown in **Figures 6A**–**G**.

### Decreased Expressions of PD-L1, T_reg_, PD-1^+^ CD8^+^ T Cells, PD-1^+^ CD4^+^ T Cells, and PD-1^+^ T_reg_ Cells and Increased CD8 and CD68 Expressions Are Associated With Better Prognosis of CC

**Figure 7:**
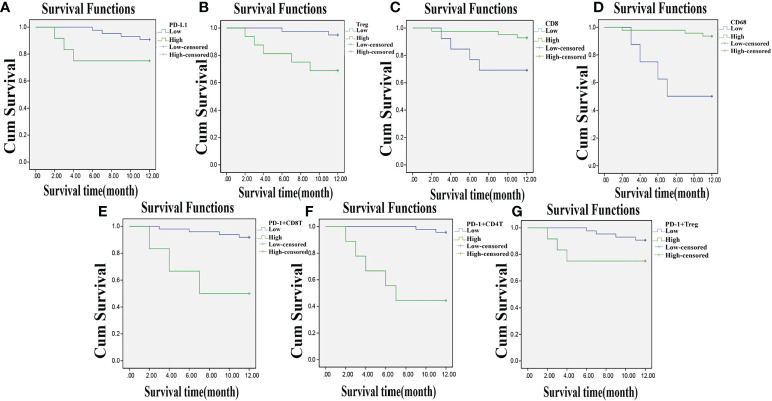
Lowly expressed PD-L1, T_reg_, PD-1^+^ CD8^+^ T, PD-1^+^ CD4^+^ T, and PD-1^+^ T_reg_, and highly expressed CD8 and CD68 are related to higher one-year OS. **Panels A, B, E–G**, the Kaplan-Meier estimator shows that low expression of PD-L1, T_reg_, PD-1^+^ CD8^+^ T, PD-1^+^ CD4^+^ T, and PD-1^+^ T_reg_ is related to high OS of CC patients; **Panels C, D**, the Kaplan-Meier estimator shows that high expression of CD8, CD68 is related to high OS of CC patients; compared with before CCRT; OS, overall survival; CC, cervical cancer; PD-L1, programmed death ligand 1; PD-1, programmed death 1.

**Figure 8:**
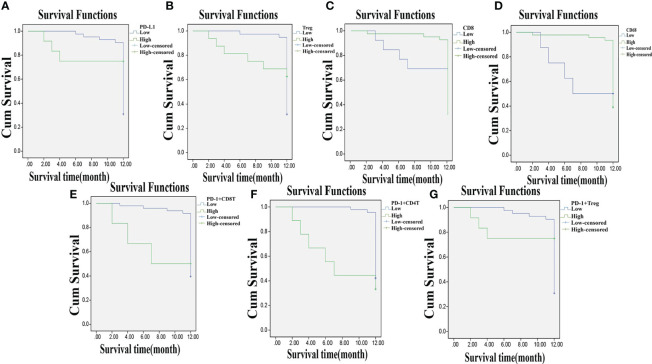
Lowly expressed PD-L1, T_reg_, PD-1^+^ CD8^+^ T, PD-1^+^ CD4^+^ T, and PD-1^+^ T_reg_, and highly expressed CD8 and CD68 are associated with DFS of CC patients. **Panels A, B, E–G**, the Kaplan-Meier estimator shows that low expression of PD-L1, T_reg_, PD-1^+^ CD8^+^ T, PD-1^+^ CD4^+^ T, and PD-1^+^ T_reg_ is related to high DFS of CC patients; **Panels C, D**, the Kaplan-Meier estimator shows that high expression of CD8, CD68 is related to high DFS of CC patients; compared with before CCRT; DFS, disease-free survival; CC, cervical cancer; PD-L1, programmed death ligand 1; PD-1, programmed death 1.

Among the 55 total patients, 7 died, the OS in 1 year was 87.30%, and the DFS was 58.18%. The relevance of the prognosis of clinical indexes included in the analysis is shown in **Supplementary Table S5**. The Kaplan–Meier estimator was used to draw the survival curve, and the results indicated that the prognosis was better when the patients had lower PD-L1, T_reg_, PD-1^+^ CD8^+^ T, PD-1^+^ CD4^+^ T, and PD-1^+^ T_reg_ expressions and higher CD8 and CD68 expressions. Log-rank test was used to determine the difference, and the results showed that the expressions of PD-L1, T_reg_, CD8, CD68, PD-1^+^ CD8^+^ T, PD-1^+^ CD4^+^ T, and PD-1^+^ T_reg_ were related to the OS and DFS of CC patients (*p* < 0.05) (**Figures 7A**–**G**, **8A**–**G**).

## Discussion

CC, characterized by a malignant tumor in the cervix, is the second leading cause of cancer burden among women worldwide (5). At present, there are still 30%–35% of CC patients who fail to completely recover from the treatment (18). Additionally, the conventional clinical variables, such as parametrial involvement, tumor size, and lymph node metastasis, are not enough to predict curative effects or formulate supplementary therapy for patients after surgery (7). Based on these facts, the study explored the effects of PD-1/PD-L1 expression on the diagnosis and prognosis of CC. The conclusive evidence indicated PD-1/PD-L1 as a viable biomarker for the CCRT efficacy of CC patients.

During the study, it was highlighted that PD-1 and PD-L1 expression in the peripheral blood and cancer tissues of CC was significantly lower after CCRT. PD-1, as a reporter encoded by the PDCD1 gene, can bind its two ligands PD-L1 and PD-L2 to suppress phosphorylation signaling and activation of immune cells (19). PD-1 is expressed on activated B cells and T cells as a coinhibitory receptor and is shown to cause an immune-mediated response and be implicated in tumor progression (20). Consistently, the PD-1/PD-L1 pathway has proven its potential in tumor immune evasion and growth (21). A previous study has reported findings in regard to the high expression of PD-1 in tumor-infiltrating lymphocytes, indicating that overexpression of PD-1 may be related to the inhibited proliferation and cytolytic activity of T cells (22). PD-1 has been reported to inhibit CD4^+^ and CD8^+^ T cells, but CD8^+^ T cells seemed to be more sensitive to PD-1 for irreversible inhibition by costimulation (23). In addition, a former study explained that low expressions of PD-1 promote the function and proliferation of tumor antigen-specific CD8^+^ T cells in melanoma (24). Furthermore, upregulated PD-1/PD-L1 on T_reg_ cells can strengthen the inhibitory function of CD^8+^ T-cell immune response during chronic virus infection (25).

It was discovered that expression of PD-1 and PD-L1 in the peripheral blood and cancer tissues was negatively correlated with the efficacy of CCRT and with the OS and DFS of CC patients. As exhibited during the study, lower expressions of PD-1 and PD-L1 in the peripheral blood and cancer tissues were connected with higher efficacy, OS, and DFS. A previous study has demonstrated that the expression of PD-1 and its ligands PD-L1 and PD-L2 on malignant cells are negatively related to prognosis for solid tumors and can be used as a marker for predicting prognosis (26). Yang et al. reported that the expression of PD-1 can define two different T-cell subpopulations, which is associated with patient outcome in follicular lymphoma (27). It has been reported as a reliable biomarker of HPV infection in the cervix (28). It has also revealed that soluble protein expression of PD-L1 in the peripheral blood could be used as a predictive biomarker for patients with diffuse large B-cell lymphoma (26). Additionally, a previous study has demonstrated that PD-L1 is overexpressed in solid tumor tissues and is correlated with a worse prognosis in multiple tumor types (29). Chen et al. demonstrated that long-term clinical responses appear in patients with melanoma, renal cell cancer, and nonsmall cell lung cancer while anti-PD-1- and anti-PD-L1-directed therapies were conducted (30). Furthermore, some other researchers also suggested that anti-PD-1 and anti-PD-L1 therapies may be solid treatment choices for CC patients (31). It is shown in the research conducted by Hassan et al. that PD-1 and PD-L1 expressions in whole blood are reduced during successful tuberculosis treatment (32). Similarly, a recent study identified that PD-1/PD-L1 inhibition shows a significant influence on DNA mismatch repair in CC patients (33).

In conclusion, our study provided substantial evidence that the efficacy of CCRT and the diagnosis of CC patients are associated with the expression of PD-1/PD-L1 in the peripheral blood and cancer tissue. It has been demonstrated that PD-L and PD-L1 expressions are influencing factors of CC occurrence and development. Low expressions of PD-L and PD-L1 are favorable prognostic factors for CC patients and have been shown to be of particular help in treating CC. It would be interesting to explore the concrete mechanism of PD-1/PD-L1 in the diagnosis and prognosis for CC, and further studies are needed in the future that include a larger sample size for more accurate and credible results.

## Data Availability Statement

The original contributions presented in the study are included in the article/**Supplementary Material**, further inquiries can be directed to the corresponding author/s.

## Ethics Statement

The studies involving human participants were reviewed and approved by the Institutional Review Board of Gui Zhou Provincial People’s Hospital (2016085). Written informed consent was obtained from all patients or their parents or guardians. The patients/participants provided their written informed consent to participate in this study.

## Author Contributions

Literature search and study design: HZ, ST, and CF. Data collection and analysis: QZ and XC. Sample collection: HZ and XC. Experiment: HZ, CF, QZ, and YL. Manuscript writing: HZ and YL. Paper review: HZ, ST, and YL. Figure drawing: QZ. All authors contributed to the article and approved the submitted version.

## Funding

This article was supported by the Science and Technology Foundation Project of Guizhou Health Commission (award No.: gzwjkj2022-027), Science and Technology Foundation Project of Guizhou Health Commission (award No.: gzwjkj2020-1-031), and Guizhou Science and Technology Planning project No.: Guizhou Science and Technology Foundation-ZK [2021] General 455.

## Conflict of Interest

The authors declare that the research was conducted in the absence of any commercial or financial relationships that could be construed as a potential conflict of interest.

## Publisher’s Note

All claims expressed in this article are solely those of the authors and do not necessarily represent those of their affiliated organizations, or those of the publisher, the editors and the reviewers. Any product that may be evaluated in this article, or claim that may be made by its manufacturer, is not guaranteed or endorsed by the publisher.
