# Supplementary material for: PD-1/PD-L1 Correlates With the Efficacy of the Treatment of Concurrent Chemoradiotherapy in Cervical Cancer
Source: Front Oncol. 2022 May 10;12:858164. doi: 10.3389/fonc.2022.858164 (PMC9128530; doi:10.3389/fonc.2022.858164)
Supplement: Supplementary file 1 [file Table_1.docx]

**PD-1/PD-L1 correlates to efficacy of the treatment of concurrent chemoradiotherapy in Cervical Cancer**

**Supplementary material**

**Table 1** Baseline characteristics of the included patients with CC.

| Characteristics | n | percentage |
| --- | --- | --- |
| Age (year) |  |  |
| ˂ 50 | 19 | 34.55 |
| ≥ 50 | 36 | 65.45 |
| Tumor size |  |  |
| ˂ 5 | 27 | 49.09 |
| ≥ 5 | 28 | 50.91 |
| Clinical stage |  |  |
| Stage II | 32 | 58.18 |
| Stage III | 23 | 41.82 |
| Lymph node metastasis |  |  |
| without | 38 | 69.09 |
| with | 17 | 30.91 |
| Histological grade |  |  |
| G1 | 7 | 12.73 |
| G2 | 36 | 65.45 |
| G3 | 12 | 21.82 |
| Pathological type |  |  |
| Epidermoid carcinoma | 49 | 89.09 |
| Adenocarcinoma | 1 | 1.82 |
| Adenosquamous carcinoma | 5 | 9.09 |
| HPV infection |  |  |
| Positive | 35 | 63.64 |
| Negative | 20 | 36.36 |
| menopause |  |  |
| with | 34 | 61.82 |
| without | 21 | 38.18 |
| Tumor marker |  |  |
| CEA | 25 | 45.45 |
| SCC-Ag | 30 | 54.55 |
| Gravidity and parity number |  |  |
| ≤ 3 | 21 | 38.18 |
| ˃ 3 | 34 | 61.82 |
| Parity |  |  |
| ≤ 3 | 38 | 69.09 |
| ˃ 3 | 17 | 30.91 |

Note: HPV, human papillomavirus; CEA, carcinoembryonic antigen; SCC-Ag, squamous cell carcinoma antigen; CC, cervical cancer; measurement data were expressed as percentage (%).

**Table 2** PD-L1 and T_reg_ cells and CD8 and CD68 expressions are associated with the efficacy of CCRT

| Index | CR + PR (n = 49) | SD + PD (n = 6) | *p* |
| --- | --- | --- | --- |
| PD-L1 | 8.17 ± 1.47 | 9.42 ± 1.15 | 0.042 |
| T_reg_ | 4.02 ± 1.03 | 5.27 ± 0.96 | 0.005 |
| CD8 | 12.58 ± 1.44 | 10.64 ± 1.36 | 0.042 |
| CD68 | 12.23 ± 1.28 | 10.12 ± 1.06 | < 0.001 |

Note: CR, complete response; PR, partial response; SD, stable disease; PD, progressive disease; measurement data were expressed as mean and standard deviation and analyzed by independent sampleindependent sample *t* test; PD-L1, programmed death ligand 1; CCRT, concurrent chemoradiotherapy.

**Table 3** Reduced PD-1^+^ CD8^+^ T cells, PD-1^+^ CD4^+^ T cells, and PD-1^+^ T_reg_ cells is associated with high efficacy of CCRT

| Index | CR + PR (n = 49) | SD + PD (n = 6) | *p* |
| --- | --- | --- | --- |
| PD-1^+^ CD8^+^ T cells | 2.03 ± 0.45 | 3.03 ± 1.28 | 0.042 |
| PD-1^+^ CD4^+^ T cells | 2.15 ± 0.63 | 3.29 ± 1.49 | 0.005 |
| PD-1^+^ T_reg_ cells | 0.16 ± 0.06 | 0.24 ± 0.03 | 0.008 |

Note: CR, complete response; PR, partial response; SD, stable disease; PD, progressive disease; measurement data were expressed as mean and standard deviation and analyzed by independent sample *t* test; PD-1, programmed death 1; CCRT, concurrent chemoradiotherapy.

**Table 4** PD-1/PD-L1 is effective for evaluation of efficacy of CCRT in CC patients

| Indexes | Youden’s index | Sensitivity | Specificity |
| --- | --- | --- | --- |
| PD-L1 | 0.503 | 0.667 | 0.837 |
| T_reg_ | 0.609 | 0.833 | 0.776 |
| CD8 | 0.670 | 0.837 | 0.833 |
| CD68 | 0.793 | 0.959 | 0.833 |
| PD-1^+^ CD8^+^ T | 0.626 | 0.667 | 0.959 |
| PD-1^+^ CD4^+^ T | 0.646 | 0.667 | 0.980 |
| PD-1^+^ T_reg_ | 0.650 | 0.833 | 0.816 |

Note: PD-L1, programmed death ligand 1; PD-1, programmed death 1.

**Table 5** Relevance of clinical factors with one-year OS of CC patients

| Factors | n | One-year OS | *p* | One-year DFS | *p* |
| --- | --- | --- | --- | --- | --- |
| Age (year) |  |  | 0.722 |  | 0.975 |
| < 50 | 19 | 17 (89.47) |  | 11 (57.89) |  |
| ≥ 50 | 36 | 31(86.11) |  | 21 (58.33) |  |
| Tumor size |  |  | 0.206 |  | 0.698 |
| < 4 | 27 | 22(81.48) |  | 15 (55.56) |  |
| ≥ 4 | 28 | 26(92.86) |  | 17 (60.71) |  |
| Clinical stage |  |  | 0.114 |  | 0.034 |
| Stage II | 32 | 26 (81.25) |  | 23 (71.88) |  |
| Stage III | 23 | 22 (95.65) |  | 10 (43.48) |  |
| Lymph node metastasis |  |  | 0.013 |  | 0.598 |
| No | 38 | 36(94.74) |  | 23 (60.53) |  |
| Yes | 17 | 12(70.59) |  | 9 (52.94) |  |
| Histological grade |  |  | 0.003 |  | 0.898 |
| G1 | 7 | 7(100.00) |  | 4 (57.14) |  |
| G2 | 36 | 34(94.44) |  | 22 (61.11) |  |
| G3 | 12 | 7(58.33) |  | 6 (50.00) |  |
| Pathological type |  |  | 0.821 |  | 0.661 |
| Epidermoid carcinoma | 49 | 43(87.76) |  | 29 (58.00) |  |
| Adenocarcinoma | 1 | 1(100.00) |  | 2 (50.00) |  |
| Adenosquamous carcinoma | 5 | 4(80.00) |  | 1 (100.00) |  |
| HPV infection |  |  | 0.702 |  | 0.718 |
| Positive type | 35 | 31(88.57) |  | 21 (60.00) |  |
| Negative type | 20 | 1(85.00) |  | 11 (55.00) |  |
| Menopausal situation |  |  | <0.001 |  | < 0.001 |
| Yes | 34 | 29(85.29) |  | 13 (38.24) |  |
| No | 21 | 19(90.48) |  | 19 (90.48) |  |
| Tumor marker |  |  | 0.076 |  | 0.178 |
| CEA | 25 | 24(96.00) |  | 17 (68.00) |  |
| SCC-Ag | 30 | 24(80.00) |  | 15 (50.00) |  |
| Gravidity and parity number |  |  | 0.883 |  | 0.902 |
| ≤ 3 | 21 | 17(80.95) |  | 12 (57.14) |  |
| > 3 | 34 | 31(91.18) |  | 20 (58.82) |  |
| Parity |  |  | 0.886 |  | 0.111 |
| ≤ 3 | 38 | 33(86.84) |  | 23 (60.53) |  |
| > 3 | 17 | 15(88.24) |  | 14 (82.35) |  |
| PD-L1 |  |  | 0.043 |  | 0.098 |
| Low expression | 46 | 42(91.30) |  | 29 (63.04) |  |
| High expression | 9 | 6(66.67) |  | 3 (33.33) |  |
| T_reg_ |  |  | 0.018 |  | 0.046 |
| Low expression | 39 | 37(94.87) |  | 26 (66.67) |  |
| High expression | 16 | 11(68.75) |  | 6 (37.50) |  |
| CD8 |  |  | < 0.001 |  | 0.008 |
| Low expression | 13 | 9(69.23) |  | 4 (66.67) |  |
| High expression | 39 | 39(100.00) |  | 28 (37.50) |  |
| CD68 |  |  | < 0.001 |  | 0.612 |
| Low expression | 8 | 4(50.00) |  | 4 (30.77) |  |
| High expression | 47 | 44(93.62) |  | 28 (71.79) |  |
| PD-1^+^ CD8^+^ T |  |  | 0.037 |  | 0.379 |
| Low expression | 48 | 44 (91.67) |  | 29 (60.42) |  |
| High expression | 7 | 4 (57.14) |  | 3 (42.86) |  |
| PD-1^+^ CD4^+^ T |  |  | 0.006 |  | 0.004 |
| Low expression | 47 | 44 (93.62) |  | 27 (57.45) |  |
| High expression | 8 | 4 (50.00 |  | 5 (62.50) |  |
| PD-1^+^ T_reg_ |  |  | 0.149 |  | 0.008 |
| Low expression | 43 | 39 (90.70) |  | 29 (67.44) |  |
| High expression | 12 | 9 (75.00) |  | 3 (25.00) |  |

Note: OS, overall survival; CC: cervical cancer; DFS, disease-free survival; HPV, human papillomavirus; CEA, carcinoembryonic antigen; SCC-Ag, squamous cell carcinoma antigen; PD-L1, programmed death ligand 1; PD-1, programmed death 1; measurement data were expressed as percentage (%) and analyzed by chi-square test
